# An Online Hand Exercise Intervention for Adults With Rheumatoid Arthritis (mySARAH): Design, Development, and Usability Testing

**DOI:** 10.2196/10457

**Published:** 2018-06-27

**Authors:** Cynthia Srikesavan, Esther Williamson, Tim Cranston, John Hunter, Jo Adams, Sarah E Lamb

**Affiliations:** ^1^ Rehabilitation Research in Oxford Nuffield Department of Orthopedics, Rheumatology, and Musculoskeletal Sciences University of Oxford Oxford United Kingdom; ^2^ Oxford Clinical Trials Research Unit Nuffield Department of Orthopedics, Rheumatology, and Musculoskeletal Sciences University of Oxford Headington, Oxford United Kingdom; ^3^ Centre for Innovation and Leadership in Health Sciences Faculty of Health Sciences University of Southampton Southampton United Kingdom

**Keywords:** rheumatoid arthritis, hand joints, exercise training, web-based

## Abstract

**Background:**

The Strengthening and Stretching for Rheumatoid Arthritis of the Hand (SARAH) program is a tailored, progressive 12-week exercise program for people with hand problems due to rheumatoid arthritis. The program was shown to be clinically and cost-effective in a large clinical trial and is recommended by the UK National Institute for Health and Care Excellence (NICE) guidelines for rheumatoid arthritis in adults.

**Objective:**

We have developed an online version of the SARAH program (mySARAH) to make the SARAH program widely accessible to people with rheumatoid arthritis. The purposes of this study were to develop mySARAH and to evaluate and address its usability issues.

**Methods:**

We developed mySARAH using a three-step process and gaining feedback from patient contributors. After initial development, mySARAH was tested in two iterative usability cycles in nine participants using a simplified think-aloud protocol and self-reported questionnaires. We also evaluated if participants executed the SARAH exercises correctly after watching the exercise videos included on the website.

**Results:**

A preliminary version of mySARAH consisting of six sessions over a 12-week period and delivered via text, exercise videos, images, exercise plan form, exercise calendar, and links to additional information on rheumatoid arthritis was developed. Five participants (1 male; 4 females; median age 64 years) and four participants (four females; median age 64.5 years) took part in the first and second usability testing cycles respectively. Usability issues identified from Cycle 1 such as having a navigation tutorial video and individualised feedback on pain levels were addressed prior to Cycle 2. The need for more instructions to complete the mySARAH patient forms was identified in Cycle 2 and was rectified. Self-reports from both cycles indicated that participants found the program useful and easy to use and were confident in performing the SARAH exercises themselves. Eight of the nine participants correctly demonstrated all the exercises.

**Conclusions:**

mySARAH is the first online hand exercise intervention for people with rheumatoid arthritis. We actively involved target users in the development and usability evaluation and ensured mySARAH met their needs and preferences.

## Introduction

### Background

The Strengthening And stretching for Rheumatoid Arthritis of the Hand (SARAH) program is a tailored, progressive12-week exercise program for people with hand problems due to Rheumatoid Arthritis (RA) [1]. The SARAH program was designed as an addition to best practice usual care (joint protection education and functional splinting and assistive devices) for adults with RA who had pain and hand function problems and had been on a stable drug regimen for at least 3 months. A pragmatic clinical trial [2,3] was conducted at 17 National Health Service (NHS) sites across the United Kingdom in 490 people who were randomized to receive best practice usual care or best practice usual care plus the SARAH program [3].

Patients who received the SARAH program in the trial attended 6 face-to-face appointments with a registered physiotherapist or occupational therapist who was a hand therapist or was experienced in rheumatology. The program included 7 upper limb mobility exercises—metacarpophalangeal flexion, tendon gliding (hook, straight, and full fist), radial walking, finger abduction, wrist circumduction, hand-behind-head, and hand-behind-back and 4 strength exercises for the hand—gross grip, pinch grip, finger adduction, and eccentric wrist extension.

Integral to the SARAH program are behavioral support strategies such as self-monitoring, goal setting, and action planning to improve patients’ self-efficacy, that is, the patients’ confidence to carry out the SARAH exercises independently. At the start of the program, patients were assisted by therapists to complete a personal exercise guide to set functional goals relating to their hand problems in accordance with SMART (Specific, Measurable, Attainable, Relevant, and Timely) principles and to make an exercise plan of “when” and “where” to do the SARAH exercises. Patients were also asked to complete an exercise diary to monitor the completion of the exercises [1-3].

During the subsequent appointments, the therapists and patients jointly reviewed the exercise diary and the personal exercise guide to set new goals and an exercise plan or modify them, if required. If a patient had difficulties adhering to the SARAH program, the patient and the therapist worked together to identify barriers to complete the exercises and to discuss realistic solutions and ways to maximize facilitating factors. A barriers and facilitators form was completed to guide this discussion. Exercises were progressed or regressed using a standardized protocol to ensure that the exercises were tailored to each patient. Patients were provided with a discharge advice sheet, exercise booklet and copies of exercise diary, personal exercise guide, and barriers and facilitators form during their final clinical appointment. They were encouraged to continue the exercises independently at home.

The key findings of the SARAH trial [3] were as follows. At 4 months, the group that received the SARAH program showed improvements in hand function double that of the usual care group (8.7 points improvement in the hand function subscale of the Michigan Hand Outcome Questionnaire (0-100) compared with an improvement of 4 points; mean difference 4. 7 points). At 12 months, the group that received the SARAH program had improvements in hand function double that of the usual care group (7.9 points improvement in the hand function subscale of the Michigan Hand Outcome Questionnaire compared with an improvement of 3.6 points; mean difference 4.3 points). The SARAH program did not result in any adverse effects, for example, increased joint pain, stiffness, or “flare-ups.” The SARAH program was also cost-effective.

These findings led to an update of the 2015 NICE guidelines recommending the SARAH program for adults who have hand problems due to RA [4]. Following this, it was important to develop a plan to disseminate the evidence-based SARAH program and ensure uptake by the target users, that is, people with RA and, thereby, facilitate improved patient care.

The overarching purpose of this project was to evaluate the adaptation of the SARAH program, originally designed to be delivered face-to-face by a therapist, to a self-guided online version (mySARAH), in which people with RA undertake the SARAH program without therapist supervision. We propose a knowledge translation initiative of an online version of the SARAH program (mySARAH) to disseminate the SARAH program to the target users by taking advantage of the increasing accessibility and use of the Internet [5-7].

The dissemination of mySARAH is guided by the 5 steps of Analysis, Design, Development, Implementation, and Evaluation (ADDIE) instructional system design model [8,9]. We will report on the first 3 steps of this process in this paper.

### Step 1: Analysis

A needs assessment was undertaken to understand what target users required in a knowledge translation tool (mySARAH) to bridge the gap between knowledge (SARAH program) and action (making the SARAH program available in an easily accessible format for people with RA).

### Step 2: Design

The online prototype of mySARAH was designed and users provided feedback.

### Step 3: Development

The preliminary version of mySARAH was developed and usability issues were evaluated for a final version. Future work will focus on the final 2 steps.

### Step 4: Implementation

mySARAH will be launched, first with a small group of target users and then into the public domain.

### Step 5: Evaluation

Reach, Effectiveness, Adoption, Implementation, and Maintenance of mySARAH will be measured in a small group of target users and then a large target population.

### Objectives

Step 1 consists of the needs assessment. Here our objectives were to collect users’ opinions and preferences for mySARAH and adapt the SARAH program to fit mySARAH. Step 2 involves the design of mySARAH. Here we designed the online prototype of mySARAH and collected user feedback on the prototype. Step 3 involves the development of mySARAH. Our objectives at this stage were to revise the mySARAH prototype toward the preliminary version, evaluate and address usability issues, evaluate if participants could replicate the SARAH exercises correctly, and produce the final version of mySARAH.

## Methods

**Figure 1:**
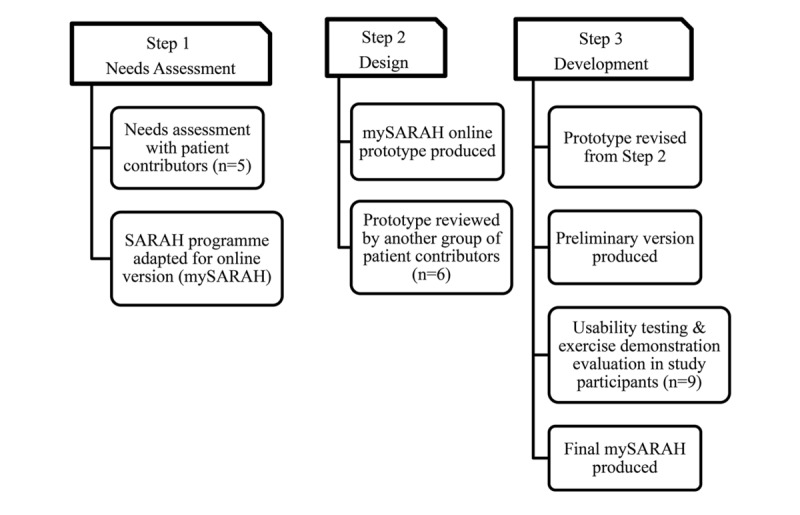
Study flow diagram.

A flow diagram of the study is presented in Figure 1.

### Step 1: Needs Assessment of mySARAH

We invited patient contributors from the public and the local branch of the National Rheumatoid Arthritis Society (patient support group) to assist with the development of mySARAH. We conducted face-to-face or phone meetings with 5 patient contributors (age range 50 to 66 years, duration range since RA diagnosis 1-12 years). We explained the components, including patient advice, the type of exercises, the number of exercise sessions, and the use of exercise diaries, goal setting and exercise planning, and how these components might be transferred to mySARAH, of the SARAH program delivered in the SARAH clinical trial. We explored their needs, preferences, and expectations for an online hand exercise program. We then collectively summarized their input. In response to this, the research team adapted the SARAH program for the mySARAH online prototype. The user feedback and SARAH program adaptations agreed upon by the team are presented in the Results section.

### Step 2: Designing mySARAH

We incorporated the common heuristic principles recommended by Baumel and Muench [10] into the mySARAH prototype. For example, simple functionality and navigation features, a tunneled approach to every exercise, and review session to respond to users’ needs with respect to the standard hand clinical appointments, “In-house” tools such as a “Contact us” button to facilitate user engagement, automatic email reminders to remind users about sessions that were missed or incomplete, and an exercise checklist and exercise calendar features for self-monitoring.

We also reviewed the published literature [11] to identify the types of features of successful internet-delivered self-guided health interventions and included them in mySARAH, for example, 1) having progressive modules over weeks or months requiring active user engagement, 2) having external links for additional health information, 3) having cognitive behavioral strategies, 4) having self-monitoring tools, and 5) providing patient education.

We further used the Behavioral Intervention Technology model [12,13] to schematically map the components of mySARAH (Table 1). The Behavioural Intervention Technology (BIT) Model summarizes the following 5 components to describe an eHealth intervention: aims, behavior change strategies, elements, characteristics, and workflow. The aims and behavior change strategies cover the conceptual aspects of “Why” and “How” and the other 3 components cover the technical aspects of “What,” “How,” and “When” of an eHealth intervention.

**Table 1:** The Behavioural Intervention Technology (BIT) Model mapped to the online version of the Strengthening And stretching for Rheumatoid Arthritis of the Hand (mySARAH) exercise intervention.

Conceptual and technical components	BIT Model components	mySARAH Examples
Why (Conceptual)	Aims	To provide adults with RA^a^ access to the SARAH^b^ program as part of a strategy for self-management To teach them to carry out the SARAH exercises correctly To promote long-term adherence to the SARAH exercises To improve and maintain hand function
How (Conceptual)	Behavioral change strategies	Knowledge: Information about RA, why hand exercises are important Goal setting: Set SMART^c^ goals related to hand function Action planning: Plan “When” and “Where” to do the SARAH exercises Problem solving: Identify and overcome barriers to exercise adherence or to maximize the use of facilitators Self-monitoring: Monitor one’s own exercise adherence behavior Review goals: Modify or set new SMART goals Instruction and Demonstration: Advice on joint protection and demonstration of SARAH exercises
What (Technical)	Elements	Reminder emails, exercise calendars, messaging, session notes, summary reports, and user fillable forms
How (Technical)	Characteristics	Medium: Text, images and videos Complexity: Easy to complete tasks, forms, and exercise calendars Aesthetics: Simple and less distractive Personalization features: None
When (Technical)	Workflow	Tunneled, Task-Based (example: user must complete mySARAH Session 1 to access Session 2), and time-based delivery (example: Successive mySARAH Sessions scheduled based on previous session completion date) Continued user access to mySARAH elements after 12 weeks

^a^RA: rheumatoid arthritis.

^b^SARAH: Strengthening And stretching for Rheumatoid Arthritis of the Hand.

^e^SMART: Specific, Measurable, Attainable, Relevant, and Timely.

The conceptual “Why” defines the clinical aims of the intervention and “How” defines the behavioral strategies used to achieve the aims. The technical “What” defines the components of the intervention, “How” describes the characteristics (the medium used, the complexity of the content, aesthetics, and personalization features) of the components, and “When” defines the workflow of the intervention delivery.

We designed the prototype on Drupal (Version 7.0), a widely used open source content management system [14]. A simple layout and a relatively plain background were used in mySARAH. A total of 6 SARAH exercise and review sessions were designed over a 12-week period where users learned to perform SARAH exercises, set their own SMART goals, and planned their exercise schedule. mySARAH included exercise calendars, a pain scale, summary reports of patient completed goals and exercise plan form, a frequently asked questions section, exercise illustrations, exercise and instructional videos, and a facility to download and save a digital copy of the content of each completed session. It also had additional patient information on RA and links to online suppliers to purchase the SARAH exercise equipment, such as gel balls, thera putty, and resistance bands. A multimedia approach with a combination of text, videos, and images was utilized for intervention delivery.

All sessions were tunnel-based, that is, users must complete a session before advancing to the next one. The consecutive session was automatically scheduled on the user’s calendar based on the completion date of the previous session. For example, upon completing session one, the second session would be released to the user after a weeks’ time.

We used the professionally produced videos on joint protection advice and SARAH exercises that were produced to train hand therapists to deliver the SARAH program using an online training program [15]. We also produced preliminary videos with a member of the team on goal setting, action planning, and simple ways to adhere to SARAH exercises.

We aimed to write the mySARAH content in plain English language without any technical or medical jargon. We checked this using the Gunning Fog index [16], a formula which estimates the readability of a piece of text by considering the number of words per sentence and the proportion of words which contain 3 or more syllables. The Fog readability index indicates the number of years of formal education required for a reader to understand the text. We used an online tool [16] to check the readability of mySARAH. In order to be readable and comprehensible to patients from a broad range of educational backgrounds, we aimed for school grades between 5 and 9 that correspond to 10 and 14 years of reading age [17].

The prototype and mySARAH logo were reviewed by another group of 6 patient contributors (age range 59-75 years, duration since RA diagnosis 1-5 years) in a half-day meeting. Following this meeting, we produced additional preliminary videos to describe patients’ experiences on how RA affected their hand function and why exercising the hands was important. The modifications made in the mySARAH prototype are presented in the Results section.

### Step 3: Developing mySARAH

Guided by Step 2, we modified the prototype and developed the preliminary version of mySARAH. We then tested mySARAH to identify and resolve usability issues for producing the final version. The usability testing protocol was reviewed and approved by the Medical Sciences Inter-Divisional Research Ethics Committee, University of Oxford (R52172/RE001).

Based on the existing evidence that over 80% of usability issues can be identified with 5-9 participants and 95% with 9 participants [18,19], we proposed a convenience sample of 10 participants for usability testing. Adults having problems with hand function due to RA and living within 2 hours of travel to the study site were considered eligible to participate in the usability testing. Participants were invited via online advertisements, e-newsletters, local patient groups, and social media of Arthritis Research UK, National Rheumatoid Arthritis Society and Patients Active in Research organizations, and by word of mouth. We asked interested volunteers to contact the SARAH implementation team directly by email or phone. Appointments were arranged for the individual volunteers to attend a one-off 90-minute usability testing session at the study site.

Two researchers from the SARAH implementation team conducted the sessions. One researcher observed and took notes, whereas the other was a session facilitator. The facilitator explained the testing procedures to each participant, emphasizing that it was the evaluation of the website and not the user. Participants were asked to provide information on their age, gender, educational level, employment status, and ethnicity, years since RA diagnosis, and hours spent on internet each day. We conducted two iterative cycles, the first cycle with 5 volunteers and the second with 4 volunteers. We used the following procedures in our usability testing.

#### Simplified Concurrent Think-Aloud Protocol

The facilitator asked participants to navigate through mySARAH, complete assigned tasks [20,21], for example, creating an account, watching videos, and completing a session, and simultaneously talk about what they feel, see, or think while browsing. When participants had difficulties verbalizing, the facilitator encouraged them by a “Keep talking” signboard and assisted with prompts only if required. The think-aloud sessions were audio-recorded.

#### Exercise Demonstration

Next, the facilitator asked participants to watch one SARAH exercise video at a time and repeat the exercise while watching. The video was then closed, and the facilitator asked them to demonstrate 3 repetitions of the exercise they had just watched. The facilitator also monitored participants for pain or discomfort in their fingers and/or wrists while demonstrating the exercises, for example, if they were stretching out a stiff joint. If a participant reported pain beyond slight discomfort, we reduced the number of repetitions from 3 to 1 or discontinued the demonstration session.

The note-taker observed the participants’ ability to correctly demonstrate each exercise, including choosing the right baseline resistance level for strength exercises, and documented any difficulties, doubts, and comments reported. A simple 1-3-point scale (1=correctly demonstrated, 2=assistance required from evaluator or by replaying the video, and 3=difficulty demonstrating the exercise correctly after being assisted), developed by the SARAH implementation team, was used to rate the correct execution of exercises and baseline load setting.

#### Subjective Reports

We used the Computer System Usability Questionnaire [22] to evaluate the user satisfaction, ease of use, information, and interface of online program with a 7-point Likert scale, 1 representing “Strongly disagree” and 7 representing “Strongly agree.” We measured the perceived usefulness with a 1-5 Likert scale that is scored from 1=Not at all useful to 5= Extremely useful; ease of use with a 1-5 Likert scale that is scored from, 1= Very difficult to 5= Very easy; and confidence in doing the SARAH exercises with a 1-5 Likert scale that is scored from 1=Not at all confident to 5=Very confident. The findings from this step are presented in the Results section.

### Data Analysis

We listened to the audio files of the think-aloud sessions along with notes from each session and created a list of key usability issues reported by participants. The demographic characteristics of usability testing participants were summarized, and the questionnaire scores were reported as medians and interquartile ranges.

## Results

### Step 1: Needs Assessment of mySARAH

The patient contributors preferred a hand exercise website having (1) a simple layout, (2) short exercise and instructional videos, (3) brief paragraphs with content written in a clear and straightforward language, (4) links to additional information on rheumatoid arthritis, (5) email reminders with option to select the frequency of reminders received, (6) a simple screening process with questions confirming age and RA diagnosis, and (7) a separate section for “Frequently asked questions.”

In addition to the above, the following modifications were made from the SARAH program in the trial for the mySARAH prototype. In the clinical trial, participants attended face-to-face appointments with a hand therapist at weeks 1, 2, 4, 6, 9, and 12. For mySARAH, we made some modifications to the timing of sessions to better fit the online delivery of the program. mySARAH sessions were scheduled to occur at weeks 1, 2, 3, 6, 9, and 12. We used a simple lay term “exercise plan form” for “personal exercise guide.” We simplified the personal exercise guide by removing the confidence scale asking patients how confident they were to achieve their goals and the exercise contract section between the patient and therapist. Instructional videos provided tips on adhering with the program instead of using the barriers and facilitators form and two mySARAH logos were designed.

### Step 2: Designing mySARAH

The analysis of the text (excluding tables, figures, hyperlinks) contained in mySARAH produced a Gunning Fog readability index ranging from approximately 7.9-11 years of education, which corresponds to an approximate reading age of 13-16 years. Some text was higher in the readability levels than ideal, but this was due to the high proportion of polysyllabic words and medical terms (examples: exercise and RA) that could not be modified further. We provided simple explanations of any medical terms.

The approximate session-wise Gunning Fog indexes were as follows: Session 1: 9.6 (reading age 15 years), Session 2: 7.9 (reading age 13 years), Session 3: 10.6 (reading age 16 years), Session 4: 10.8 (reading age 16 years), Session 5: 11 (reading age 16 years), and Session 6: 11 (reading age 16 years).

The patient contributors who participated in the half-day meeting liked the layout and look of the website. They felt that the information and the language were clear and easy to follow. They also liked the features of exercise calendar, email reminders, exercise videos, frequently asked questions section, and facility to have a summary record of their goals and exercise plan. They also agreed on one of the two mySARAH logos produced by the team. Several revisions were suggested, including breaking long paragraphs into shorter paragraphs, using bullet points to break up lengthier sections of text, ensuring the pages were not cluttered, page proofreading, the addition of contact details for exercise equipment suppliers, and additional details, at the end of the program, pertaining to a continued access to mySARAH.

### Step 3: Developing mySARAH

The preliminary version of mySARAH was produced and the revisions suggested by users in Step 2 were incorporated. The resultant version still closely resembled the prototype.

We enrolled 10 participants in the usability testing, which took place in two cycles. A total of 9 participants completed the testing and one volunteer withdrew after consenting due to a family member’s sickness.

The demographic characteristics of participants who took part in the usability testing Cycle 1 and Cycle 2 sessions are presented in Table 2. Participants felt that the mySARAH website was self-explanatory, easy to use, and contained all the information needed about SARAH. They reported that the registration process was straightforward, the goal setting and exercise planning form and exercise calendar was helpful, the website content was relevant, the exercise and other videos were helpful and engaging, and the forms were easy to fill out. The main usability issues identified by participants from each cycle and the subsequent revisions are listed in Table 3.

The Computer System Usability Questionnaire and Likert Scale scores from both cycles of usability testing are listed in Table 4. All participants had a good agreement (with scores above 6) on almost all the items of the Computer System Questionnaire, especially in terms of satisfaction, ease of use, and the content. Overall, 3 participants found Item 8 stating “I believe I became productive quickly using this system” irrelevant to the tasks they completed and, therefore, did not score it, and 7 participants felt that Item 9 stating “The system gives error messages that clearly tell me how to fix problems” was not relevant because they did not encounter any issues while filling in the fields in mySARAH forms. Therefore, we have not reported these two items. However, participants raised the concern of being notified to rectify any errors while filling the forms. We duly addressed this usability issue by adding pop-up error notifications in the final version.

The Likert scale scores indicate that all users found the program useful and easy to use and were confident in their ability to do the SARAH exercises themselves.

Overall, majority of the participants (8/9, 89%) correctly demonstrated all 11 SARAH exercises, scoring 1 on the 1-3 exercise demonstration scale. One participant required guidance for the “Spread fingers” and “Hand squeeze” exercises and found holding the resistance band between fingers for the “Wrist backward bends” strength exercise difficult to demonstrate.

After addressing Cycle 2 usability issues, a few additional revisions were made by the research team in the final version of mySARAH. Specifically, a “Go to homepage” tab was created to signpost to respective sessions on logging in. We produced and filmed a professional mySARAH promotional video, a navigation tutorial video, and informational videos on clinical aspects of RA and behavioral strategies for exercise adherence. A patient video demonstrating how to set baseline load for each strengthening exercise was additionally produced (Multimedia Appendix 1). Both therapist -patient and patient-demonstrated videos for wrist backward bends exercise were combined as a single video and added to mySARAH. We had the mySARAH pages proofread by a patient volunteer and a member of the research team.

### Final version of mySARAH

**Figure 2:**
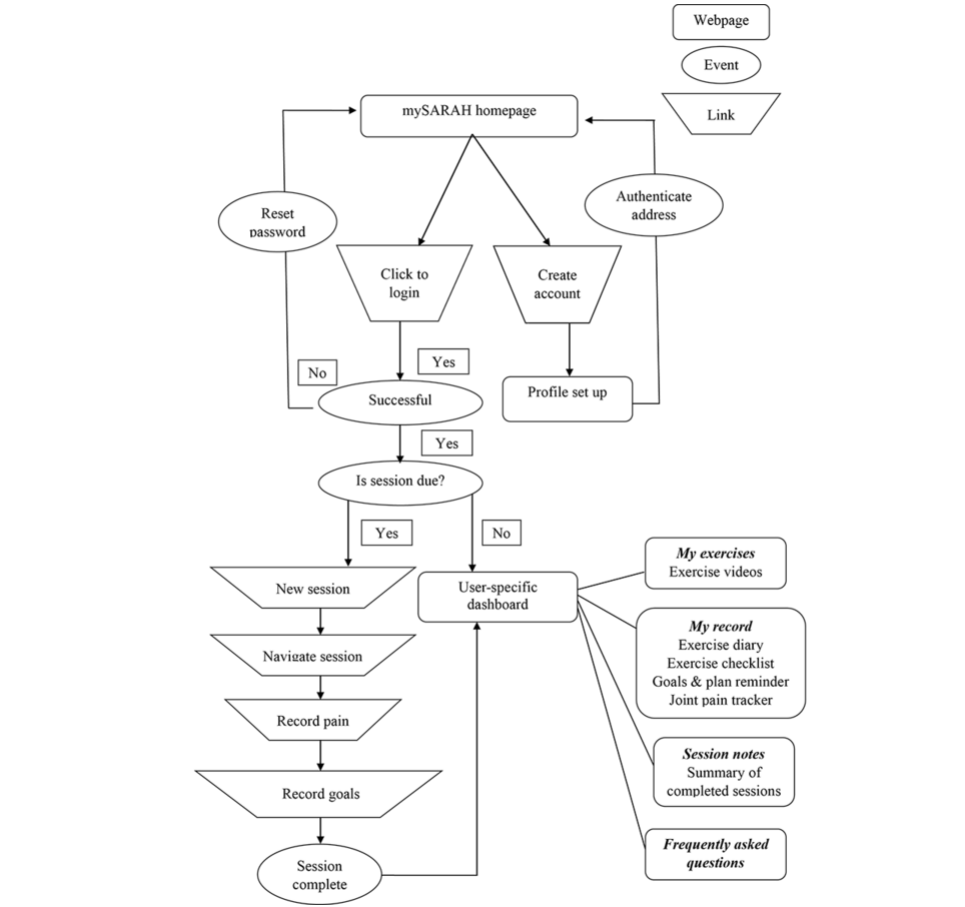
Navigation workflow of mySARAH.

A brief description of mySARAH (Multimedia Appendix 1) and screenshots of mySARAH (Multimedia Appendix 2) are presented herein. Table 5 provides an overview of the final mySARAH sessions’ content. Sessions were accessed by users on a preset timetable, so they had adequate time to perform the exercises between sessions. Figure 2 shows the navigation pathway of mySARAH.

**Table 2:** Characteristics of participants taking part in mySARAH usability Cycles 1 and 2.

Characteristics		Cycle 1	Cycle 2
Number of participants (N=9)		5	4
Age (years), median (IQR^a^)		64 (60-66)	64.5 (55-70.5)
Male/Female		1/4	0/4
**Educational qualification**			
	High school	1	1
	Bachelor	1	2
	Doctoral	3	0
	Certificate course	0	1
**Ethnicity**			
	White British, n (%)	4 (80)	4 (100)
	Indian n (%)	1 (20)	0 (0)
**Employment status n (%)**			
	Retired	3 (60)	2 (50)
	Part-time	1 (20)	1 (25)
	Full-time	1 (20)	0 (0)
	Not working	0 (0)	1 (25)
Duration since diagnosis (years), median (IQR)		17 (12-25)	12 (4-26)
Internet use/day (hours), median (IQR)		3 (2-6)	1 (1-1.5)

^a^IQR: interquartile range.

**Table 3:** Main usability issues and rectifications made in the online version of the Strengthening And stretching for Rheumatoid Arthritis of the Hand (mySARAH) exercise intervention.

Usability issues		Rectifications
**Cycle 1 (n=5)**		
	0-10 numerical pain scale was not clear	Verbal descriptors were added to the 0-10 pain scale
	The background color was very plain	A pleasant blue background was added
	A feedback report on the pain levels would be helpful	A graph feature to provide a visual feedback on the pain levels recorded during every session was added
	There was no separate section for email reminders	A separate section with an option for selecting the frequency of reminders was added
	A separate patient video demonstrating wrist backward bends exercise would be helpful	An additional patient video was added
	The size of some images was too small	Small images were increased in size
	The progression bar across sessions was not noticeable	Progression bar was increased in width
	Some pages were too wordy	Bullet points were used
	A navigation tutorial video would be helpful	A preliminary navigation tutorial video was added
**Cycle 2 (n=4)**		
	The instructions for completing forms/exercise calendar was not adequate	Clear instructions for completing forms/exercise calendar were added
	There was too much scrolling in some pages	Page screen spaces were managed to reduce scrolling
	Try more colors with pages: add 1 or 2 images on the landing page	A welcome image was added on the landing page
	How do you know if a mistake was made on a form?	A pop-up message was set up to notify any omission or error prior to submission

**Table 4:** Questionnaire scores of the usability testing (N=9) for the online version of the Strengthening And stretching for Rheumatoid Arthritis of the Hand (mySARAH) exercise intervention.

Questionnaire		Cycle 1	Cycle 2
**Computer System Usability Questionnaire items on 1-7 scale^a^, median (IQR^b^)**			
	1) Overall, I am satisfied with how easy it is to use this system	6.5 (6-7)	6.5 (6-7)
	2) It was simple to use this system	6 (6-7)	6.5 (6-7)
	3) I can effectively complete my work quickly using this system	5.8 (5.1-6.3)	6 (6-6.3)
	4) I am able to complete my work quickly using this system	5.8 (5.1-6.3)	6 (6-6.3)
	4) I am to efficiently complete my work using this system	6.5 (5.5-7)	6 (6-6)
	6) I feel comfortable using this system	6 (6-7)	7 (6.8-7)
	7) It was easy to learn to use this system	6 (6-7)	6.5 (6-7)
	8) I believe I became productive quickly using this system	N/A^c^	N/A
	9) The system gives error messages that clearly tell me how to fix problems	N/A	N/A
	10) Whenever I make a mistake using this system, I recover easily and quickly	6 (5-6.5)	6 (5.3-6)
	11) The information (such as online help, on-screen messages, and other documentation) provided with this system is clear	6.5 (5.5-7)	7 (7-7)
	12) It is easy to find the information I needed	6 (6-7)	6.5 (6-7)
	13) The information provided for the system is easy to understand	6 (6-7)	7 (6.8-7)
	14) The information is effective in helping me complete the tasks and scenarios	6 (6-7)	7 (6.8-7)
	15) The organization of information on the system screens is clear	6 (6-7)	6.5 (6-7)
	16) The interface of the system is pleasant	6 (6-7)	6 (6-7)
	17) I like using the interface of this system	6.5 (5.8-7)	6 (5.8-6.3)
	18) This system has all the functions and capabilities I expect it to have	6.5 (5.8-7)	6 (5.5-6.3)
	19) Overall, I am satisfied with this system.	6.5 (6-7)	6 (6-6.3)
**Likert scale of perceived usefulness, ease of use, and confidence, median (IQR)**			
	On a scale of 1-5, with 1 representing “Not at all useful” and 5 representing “Extremely useful,” how would you rate the overall usefulness of mySARAH?	5 (4-5)	5 (4.8-5)
	On a scale of 1-5, with 1 representing “Very difficult” and 5 representing “Very easy,” how would you rate the overall ease of use of mySARAH?	4 (3-4)	4.5 (4-5)
	On a scale of 1-5, with 1 representing “Not at all confident” and 5 representing “Very confident,” how would you rate your confidence in doing the SARAH^d^ exercises by yourself?	4.5 (4-5)	5 (5-5)

^a^1=strongly disagree, 2=disagree, 3=somewhat disagree, 4=neither, 5=somewhat agree, 6=agree, 7=strongly agree.

^b^IQR: interquartile range.

^c^N/A: not applicable.

^d^SARAH: Strengthening And stretching for Rheumatoid Arthritis of the Hand.

**Table 5:** Outline of mySARAH sessions’ content.

Session	Suggested week of completion	Outline of content
1	Week 1	Users fill out demographic information and hand function questionnaire Users rate the pain in their hands on a 0-10 numerical scale Information is provided about the clinical aspects of RA^a^ and its management The SARAH^b^ mobility exercises are introduced Users are taught how to set SMART^c^ goals and plan when and where to complete exercises. Users are encouraged to complete the mobility exercises daily from this point onwards
2	Week 2	The SARAH strength exercises are introduced Users are taught baseline setting for strength exercises Users review and update their goal and plan at the end of each session from this point onwards
3	Week 3	The session covers how and when users should adjust their exercises if they: Are finding them too challenging Need to make them harder
4	Week 6	The session encourages users to consider any barriers to completing their exercises, which have become apparent since beginning the program It also asks users to think about how they have overcome barriers and what else they could do in the future
5	Week 9	The session discusses the challenges to adhere to the program in the long-term Users are taught how to restart the program if they need to stop for any reason
6	Week 12	The session focuses on the continuation of the exercises after completion of the program. Users are encouraged to continue to access the resources on the website if they need to Users complete the Michigan hand function subscale, Global Rating of Change scale to measure their progress

^a^RA: Rheumatoid arthritis.

^b^SARAH: Strengthening And stretching for Rheumatoid Arthritis of the Hand.

^c^SMART: Specific, Measurable, Attainable, Relevant, and Timely.

## Discussion

### Principal Findings

The objectives of this study were to develop an online version of the evidence-based SARAH hand exercise program in collaboration with patients with RA (target users of mySARAH) and to identify and address its usability issues for a refined final version.

In general, patient contributors wanted a simple, less-cluttered, and less-wordy website. They were pleased with the purpose and content of the mySARAH prototype and found it a useful resource for people with hand function difficulties due to RA. Usability testing session participants also found mySARAH a useful and easy to use online exercise program and were confident to execute the SARAH exercises and set their baseline load for strength exercises on their own.

### End User Involvement

We wanted to develop a user-centered mySARAH website and, hence, involved patient contributors in the initial phases of developing mySARAH. This is one of the very few web-based systems that were formally tested in adults with RA [21,23]. Users are expected to learn the SARAH exercises correctly, do them daily, and progress or regress the dosage according to their capabilities because mySARAH is self-directed in nature. Therefore, in addition to the usability testing of the website, we captured how far people managed to learn and perform the exercises correctly and set their baseline load for each strength exercise. We believed that evaluating this at these earlier stages would inform whether participants found the exercise demonstration videos easy to follow and replicate them, as intended.

### Strengths of mySARAH Program

RA is a chronic condition; therefore, long-term adherence is required to maintain joint mobility and muscle strength. From the findings of the SARAH trial, we know that participants found it challenging to continue the exercises long-term, and by 2 years follow-up, many ceased their exercises [24]. One of the major strengths of mySARAH is that it will allow users continued access to the SARAH exercises from home without the need for hospital appointments and, thus, overcoming practical problems such as transportation difficulties or lack of availability of hand therapy appointments. It would also give them access to videos and information if they need reminding of the exercises following a break. They can continue to use the exercise calendar to potentially promote long-term adherence to the program. The other strength is that the program was built upon a theoretical model incorporating heuristics principles toward a user needs-based intervention.

### Limitations

This study has some limitations. Due to resource and time restraints, we did not transcribe the think-aloud sessions or code their content. The exercise demonstration evaluation scale used in this study was not a validated scale, but this evaluation was done by experienced physiotherapists and the scale was designed as a simple and pragmatic tool to record the evaluation. We intended to include people from a wide range of educational and computer literacy backgrounds. However, the majority of the participants (8/9, 89%) were white British women, and most of them (6/9, 67%) had Bachelor to Doctoral levels of formal education. The median number of years since participants were diagnosed with RA was greater than 10 years in both the usability cycles. Therefore, we are uncertain whether men, people from other cultural or educational backgrounds, or those diagnosed more recently would offer a different perspective on mySARAH. Our sample size still meets the recommended number for testing usability issues.

### Next Steps

We plan to carry out further testing in a proof-of-concept study to establish if people with RA are willing and able to complete the mySARAH program, do the exercises correctly, and undertake daily exercises

### Conclusions

Involving target users in the development process ensured that mySARAH resulted in a user-centered and user-friendly online exercise resource. The results from the usability testing show mySARAH to be an efficient and user-friendly program.

## Appendix

Multimedia Appendix 1 Brief description of mySARAH.

Multimedia Appendix 2 mySARAH Screenshots.

## Abbreviations

ADDIE: Analysis, Design, Development, Implementation, and Evaluation
BIT model: Behavioural Intervention Technology Model
mySARAH: Online version of the Strengthening And stretching for Rheumatoid Arthritis of the Hand exercise intervention
NHS: National Health Service
NICE: National Institute for Health and Care Excellence
RA: Rheumatoid Arthritis
SARAH: Strengthening And stretching for Rheumatoid Arthritis of the Hand
SMART: Specific, Measurable, Achievable, Relevant, and Timely
