# Supplementary material for: An Online Hand Exercise Intervention for Adults With Rheumatoid Arthritis (mySARAH): Design, Development, and Usability Testing
Source: J Med Internet Res. 2018 Jun 27;20(6):e10457. doi: 10.2196/10457 (PMC6041557; doi:10.2196/10457)
Supplement: Multimedia Appendix 1 [file jmir_v20i6e10457_app1.pdf]

Multimedia Appendix 1: Brief description of mySARAH

The **main landing page or the homepage** contains a welcome image, an informational video, and buttons to register or log in mySARAH. If it is the first visit, users will need to choose the option to register. Selecting this option will take them to a new page where they input their name, email address, and opting for email reminders from mySARAH. Once the account has been created, a link will be sent to authenticate user's registration and to set a password. If a user has already registered, they log in via the **Login** tab using their e-mail or username and password.

The **About** tab contains information on the programme and its development and how to register with mySARAH. The **Privacy** tab explains the accessibility and cookies policy of the website. The **Contact Us** page allows users to send a message directly to the mySARAH team. The **My Account** tab allows users to edit their personal and contact details or to set a new password.

When a user completes a session, the date for the next session will be automatically set and users will find the highlighted date on their mySARAH exercise calendar. The user will not be able to access the next session until that date. When users log in to mySARAH between sessions they will arrive on a user-specific page that has four tabs: 1. **My record**, 2. **My Exercises**, 3. **Session Notes**, and 4. **Frequently Asked Questions.**

My Record allows users to track their progress through the programme by recording their completion of the exercises on the mySARAH exercise calendar. It shows the exercise checklist for that day as well as their current SMART goal and their plan of where and when

they will complete their exercises. As the exercises on the checklist are ticked off, the corresponding date on the exercise calendar will change from red (not started) to orange (started but not finished), to green (completed).

My Exercises contains the video demonstrations and written instructions for each exercise.

Session Notes contains downloadable summaries of each session together with printable versions of each form used in the programme. The session notes and forms become available once the corresponding session has been completed.

The **Frequently Asked Questions** section relates to both the programme and to the website itself. Sample questions include: “What if I forget to do the exercises?” and “What if a button isn’t working/I think I’ve found a bug?”
