# Supplementary material for: An Online Hand Exercise Intervention for Adults With Rheumatoid Arthritis (mySARAH): Design, Development, and Usability Testing
Source: J Med Internet Res. 2018 Jun 27;20(6):e10457. doi: 10.2196/10457 (PMC6041557; doi:10.2196/10457)
Supplement: Multimedia Appendix 2 [file jmir_v20i6e10457_app2.pdf]

mySARAH

RA Hand Exercise Website

Homepage

mySARAH RA Hand Exercise Website

Home | About | Privacy | Contact Us | Login

Welcome to mySARAH

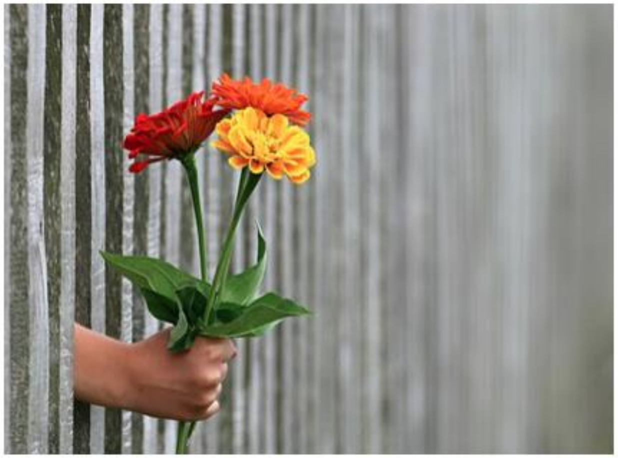

Strengthening And Stretching For Rheumatoid Arthritis of the Hand (SARAH) programme.

"When I was first diagnosed rheumatoid arthritis, I didn't have any control over it whatsoever, whereas now I'm actually doing something that's practical that might help, so the SARAH Programme for me I think has been really quite valuable because it's empowered me!" -A patient who received SARAH exercises.

Watch this video to know about mySARAH programme:

A page from Session 2

34%

Getting started on the strengthening exercises

Now, we are going to get started on the 4 strengthening exercises. You will need the equipment we told you about in the previous session – resistance band and putty.

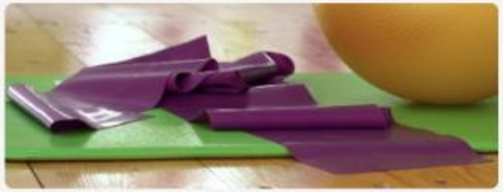

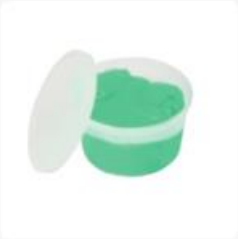

The SARAH strengthening exercises are:

1. Hand squeeze
2. Squeeze fingers
3. Finger pinch
4. Wrist backward bends

‹ Go Back   Continue ›

Pain scale

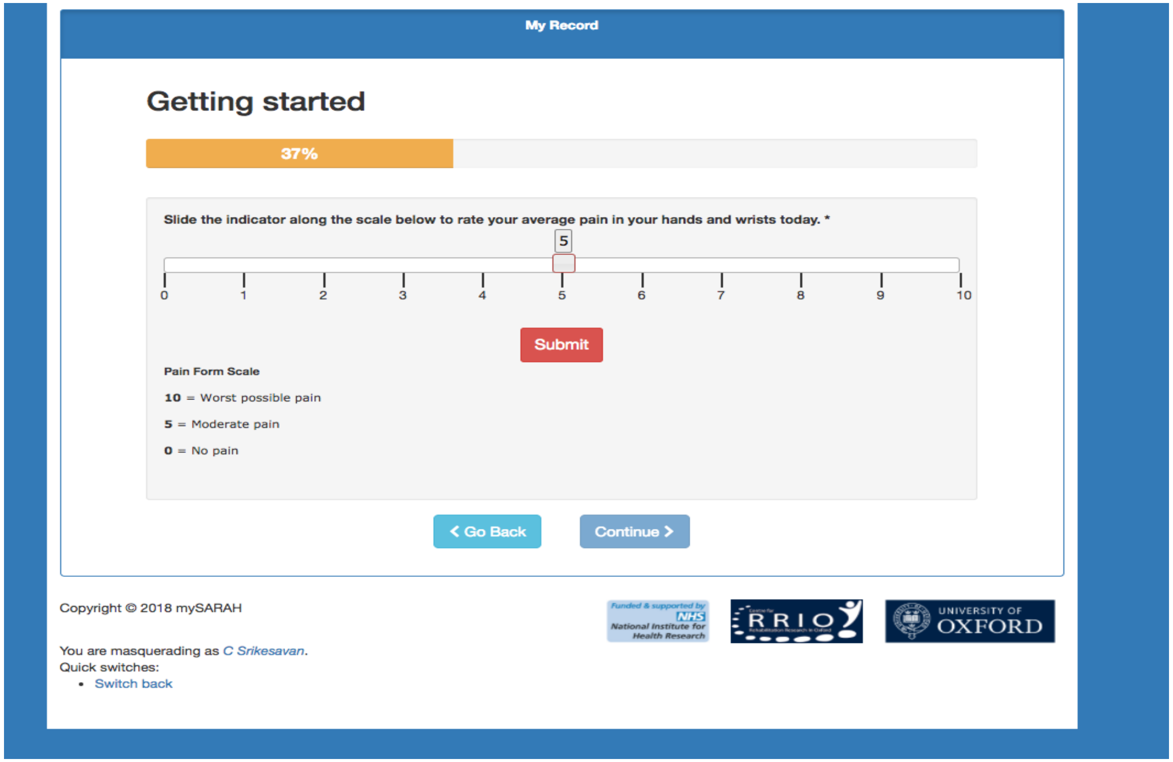
My Record
Getting started
37%
Slide the indicator along the scale below to rate your average pain in your hands and wrists today. *
5
0 1 2 3 4 5 6 7 8 9 10
Submit
Pain Form Scale
10 = Worst possible pain
5 = Moderate pain
0 = No pain
Go Back
Continue
Copyright © 2018 mySARAH
You are masquerading as C Srikesavan.
Quick switches:
Switch back
Funded & supported by NHS National Institute for Health Research
RRIO
UNIVERSITY OF OXFORD

Exercise plan form

My Exercise Plan

Please complete your exercise plan and submit.

My goal is *

For example: Write without pain

I will exercise during *

For example: My lunchtime

I will do my exercises at *

For example: My kitchen table

Submit

❮ Go Back   Continue ❯

Copyright © 2018 mySARAH

Funded & supported by NHS National Institute for Health Research

Centre for RRIO Rehabilitation Research in Oxford

UNIVERSITY OF OXFORD

Exercise calendar

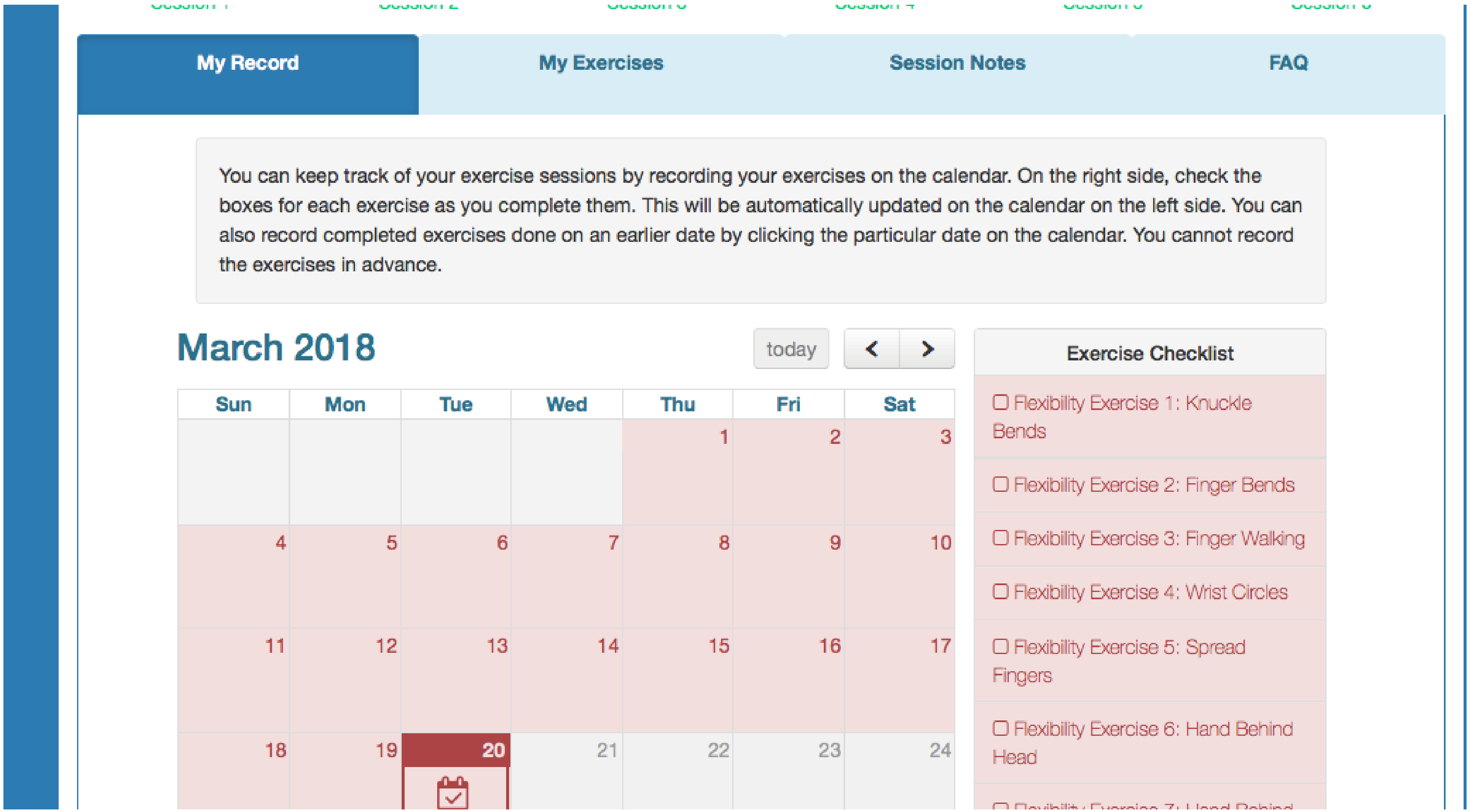

Goal reminder and Pain report

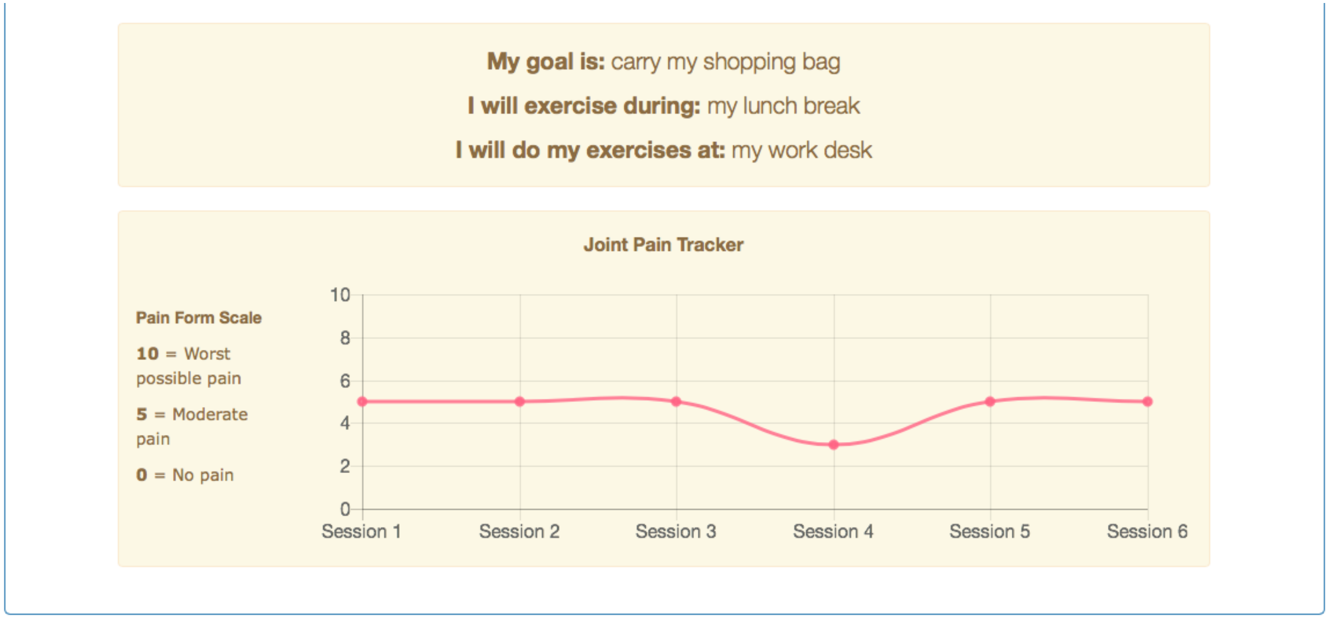

An exercise video

View Edit

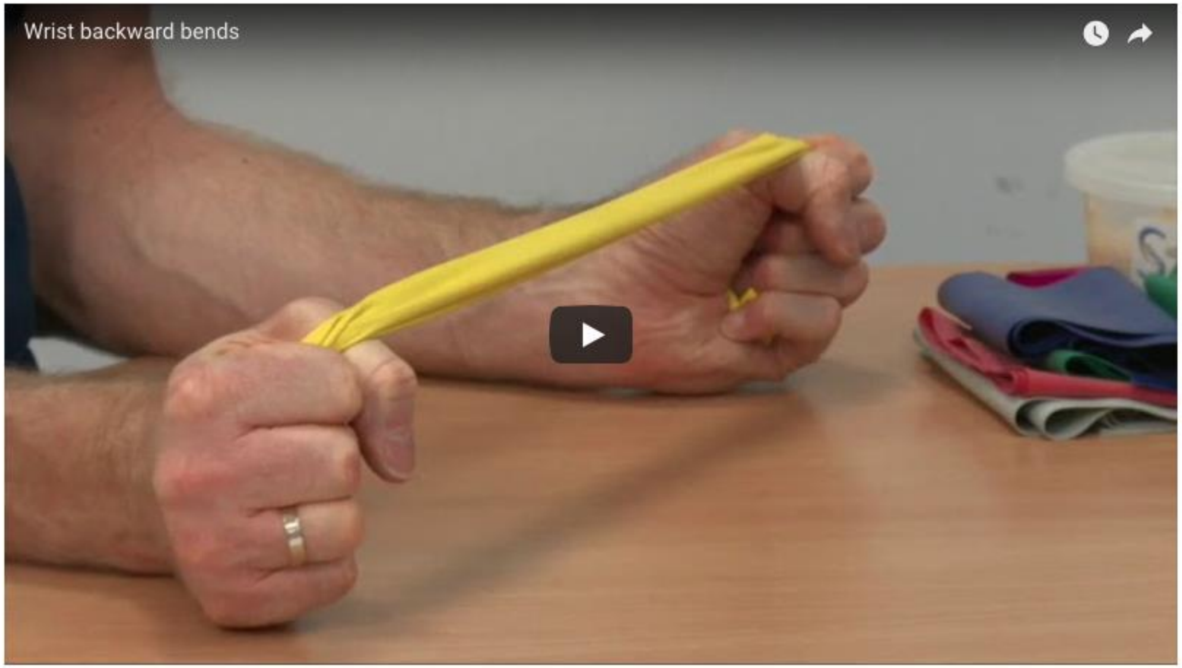

Strength Exercise 4: Wrist backward bends (Session_2)

1. Sit with elbows tucked into your side and bent to 90°.
2. Hold the elastic band in both hands with the wrists bent inwards. Make sure your arms are far enough apart so there is no slack in the elastic band.
3. Keeping your forearms still, bend your wrists backward stretching the band as much as comfortably possible.

My Record | My Exercises | Session Notes | FAQ

Session Notes

A summary of each session is available as you progress through each session. These summary can be downloaded for your own personal use.

Before you get started
Information about the equipment that you need to do the SARAH programme

Session 1
In Session 1 we talk about the management of rheumatoid arthritis including joint protection, splints and exercise. We got started on the SARAH mobility exercises. This session also covered goal setting and planning where and when to do your exercises.

Session 2
In Session 2 we started the SARAH strengthening exercises. We taught you how to select the starting point for each exercise (baseline). We reviewed your goal and your exercise plan.

Session 4
In session 4 we reviewed the steps to progress or adjust your exercises. We asked you to think about things that may be stopping you from exercising regularly as well as things that help you to exercise. We reviewed your goal and your exercise plan.

Session 5
In Session 5 we reminded you about how to progress or adjust your exercises. We talked about the challenges to sticking with the exercises long term that patients have told us. We gave you tips on restarting your SARAH exercises if you have a break from them. We reviewed your goal and your exercise plan.

mysarah.octru.ox.ac.uk/sites/default/files/session_notes/Before%20you%20get%20started.pdf" in a new tab

Frequently Asked Questions

Frequently Asked Questions

Do I need to do all SARAH exercises at the same time?

I am worried that I am not doing the exercises correctly

I am worried that I could damage my joints

What if I have severe joint pain/a flare up?

How will I find the time to do my exercises?

What if I forget to do the exercises?

Can I omit any SARAH exercise?

Is there someone I could contact if I have any questions on the SARAH programme?

What do I do if I can't play a mySARAH video?

What if a button isn't working/I think I've found a bug?

Do I have to login for all mySARAH online sessions? How many sessions do I need to complete?
